# Identification of the Genetic Variation and Gene Exchange between *Citrus Trifoliata* and *Citrus Clementina*

**DOI:** 10.3390/biom8040182

**Published:** 2018-12-19

**Authors:** Tian-Jia Liu, Jing-Jing Zhou, Fa-Yi Chen, Zhi-Meng Gan, Yong-Ping Li, Jin-Zhi Zhang, Chun-Gen Hu

**Affiliations:** Key Laboratory of Horticultural Plant Biology (Ministry of Education), College of Horticulture and Forestry Science, Huazhong Agricultural University, Wuhan 430070, China; liu.tianjia@webmail.hzau.edu.cn (T.-J.L.); hupodingxiangyu@mail.hzau.edu.cn (J.-J.Z.); chenfayi@webmail.hzau.edu.cn (F.-Y.C.); zhimenggan@webmail.hzau.edu.cn (Z.-M.G.); yplee614@webmail.hzau.edu.cn (Y.-P.L.)

**Keywords:** citrus, gene exchange, genetic variation, grafting, RNA sequencing

## Abstract

To identify the genetic variation between *Citrus trifoliata* and *Citrus clementina*, we performed genome resequencing on the two citrus species. Compared with the citrus reference genome, a total of 9,449,204 single-nucleotide polymorphisms (SNPs) and 846,615 insertion/deletion polymorphisms (InDels) were identified in the two citrus species, while 1,868,115 (19.77%) of the SNPs and 190,199 (22.47%) of the InDels from the two citrus species were located in the genic regions. Meanwhile, a total of 8,091,407 specific SNPs and 692,654 specific InDels were identified in the two citrus genotypes, yielding an average of 27.32 SNPs/kb and 2.34 InDels/kb. We identified and characterized the patterns of gene exchanges in the grafted citrus plants by using specific genetic variation from genome resequencing. A total of 4396 transporting genes across graft junctions was identified. Some specific genetic variation and mobile genes was also confirmed by Sanger sequencing. Furthermore, these mobile genes could move directionally or bidirectionally between the scions and the rootstocks. In addition, a total of 1581 and 2577 differentially expressed genes were found in the scions and the rootstocks after grafting compared with the control, respectively. These genetic variations provide fundamental information on the genetic basis of important traits between *C. trifoliata* and *C. clementina*, as the transport of genes would be applicable to horticulture crops.

## 1. Introduction

Grafting has been used in a number of plant species, including fruits, vegetables, and ornamentals, for over 2000 years of history to improve plant production, water-use efficiency, and other fruit quality traits, as well as deal with abiotic stress [1,2]. Grafting is employed to fuse material from two different plants: a part of a plant (scions) is joined to another plant (rootstocks) for the two parts to grow together [3]. Therefore, when a graft takes successfully, the rootstock and scions appear to grow as a single unit in cultivation. Graft compatibility between the scions and rootstocks is a key factor affecting the success rate of grafting. The formation of callus tissue at the graft interface and the establishment of a functional vascular system are essential prerequisites in grafted plants [2]. Grafting is a common means to clonally propagate desirable scions in many fruit trees, because the seeds from these plants cannot reproduce the original cultivar due to genomic heterogeneity [4]. The mechanisms behind the physiological interactions between rootstocks and scions in fruit trees have been studied for some time, including anatomical investigations, nutrient transport, and hormonal movements [1,5,6]. However, the molecular mechanisms of the rootstock–scion interaction are still largely unknown. Understanding the interactions between scions and rootstocks will certainly allow the exploitation of new useful germplasm and new agronomic applications in the future.

Recently, many transcriptome and proteome studies have investigated the rootstock–scion interaction in various plants that occur at the early phase in response to grafting [7,8,9]. For example, expression profiling has provided new insights into the grafting-responsive genes that are involved in many different biological and metabolic processes in watermelon [8]. In grapevine, grafting induced transcriptional changes related to various processes, including cell wall synthesis, phloem and xylem development, hormone signaling, and secondary metabolism [10]. A proteomic study revealed that the higher expressions of proteins were involved in many key biological processes such as reactive oxygen species (ROS) defense, hormonal biosynthesis, and signal transduction [11]. In Hickory, a comparative proteomic analysis showed that the flavonoid biosynthesis pathway and starch and sucrose metabolism were both significantly up-regulated in the graft unions [12]. In the last decade, it has been established that minerals, amino acids, carboxylic acids, ions, phenylpropanoids, and hormones can be exchanged between rootstocks and scions, because grafting connects the vascular tissues between rootstocks and scions [1,5,13]. Many developmental phenomena were induced in graft union by long-distance transmissible substances via phloem and xylem such as flowering, tuber formation, nodule formation, leaf development, lateral shoot formation, and defense [13,14,15,16,17,18]. Recent advances have revealed that some proteins, genes, and microRNAs (miRNAs) are transported over a long distance through the sieve tube, in order to function at sites where they are required [19,20,21].

Citrus is one of the most important fruit trees in the world. To affect citrus trees’ vigor, size, precocity, fruit quality, taste, and harvestable yield, the use of rootstocks is very popular in citrus [3,5]. To obtain insight into the potential mechanisms underlying the influence of rootstock on scion growth in citrus, Liu et al. [22] concluded that different rootstocks significantly affected the expression of the genes that are involved in the auxin signal transduction pathway and gibberellins (GA) biosynthesis pathway in the grafted plants of ‘Shatangju’ mandarin. He et al. [23] studied the mechanism between compatible and incompatible graft combinations of *Citrus grandis* (Linnai), and found that a large number of genes were involved in carbohydrate metabolism, energy metabolism, amino acid metabolism, and plant hormone signal transduction. However, the underlying molecular and genetic mechanisms for how the graft partners interact with each other to produce a successful graft remain largely unknown. The recent release of the citrus genome dramatically enhanced the efficiency of grafting research in citrus [24]. Here, we resequenced the genome of trifoliate orange (*Citrus trifoliata*) and Clementine mandarin (*Citrus clementina*), and a large number of putative genetic variations were identified based on the citrus reference genome. The distribution and predicted function of these genetic variations were analyzed in detail. To evaluate this genetic variation dataset, some genetic variations were amplified and sequenced using the Sanger method. Using specific genetic variations derived from *Citrus trifoliata* and *C. clementina* as markers, we identified the patterns of genome-wide gene exchanges between *C. trifoliata* and *C. clementina*, and provided insights into how genome-wide gene exchanges between scions and rootstocks may contribute to the genetic success of grafted plants.

## 2. Materials and Methods

### 2.1. Plant Materials

To detect gene exchanges between scions and rootstocks, a species-graft system, in which *C. trifoliata* was the rootstock and *C. clementina* was the scions, was used to identify the genes that moved across the graft union. All of the plants were grown in the greenhouse of the National Citrus Breeding Center at Huazhong Agricultural University (Wuhan, China) at a temperature of 25 ± 1 °C with 60–70% relative humidity. The juvenile material from *C. trifoliata* was obtained from the seeds of the adult mother plants. Since the embryo originates from a nuclear cell, the seedlings have the same genetic background as the mother plants. Seeds of *C. trifoliata* were sown in 20-cm pots containing commercial potting mix and perlite (3:1, *v*/*v*). Following germination, the seedlings were watered regularly with a nutrient solution. The rootstock trial contained three replicates of grafts involving three scions and three rootstocks grown in the greenhouse. The scions were grafted to six-month-old rootstocks. In this study, the scions from *C. clementina* were grafted to six-month-old *C. trifoliata* as an experimental grafting system (Supplementary Figure S1). Grafted trees were grown in a nursery for four months (Supplementary Figure S1). Only samples from mature healthy fully expanded leaves and stems (grafting union above and below 5 cm) were collected from graft unions at four months after grafting. A mixture sample taken from same rootstocks and scions before grafting was used as the control. All of the materials were collected from three individual plants for RNA extraction. All of the plant tissues were sampled according to the demands of each experiment; then, they were immediately frozen in liquid nitrogen, and stored at −80 °C until used.

### 2.2. DNA Isolation and Genome Resequencing

The plant DNA of all of the material was isolated from the leaf according to the cetyltrimethyl ammonium bromide method (CTAB) [25]. For resequencing the genome, the DNA was randomly sheared. After electrophoresis, DNA fragments of the desired length were gel-purified. Adaptor ligation and DNA cluster preparation were performed and subjected to the Solexa sequencing using an Illumina Genome Analyzer IIx (Solexa, San Diego, California, USA) [26]. Low-quality reads (<20), reads with adaptor sequences, and duplicated reads were filtered, and the remaining high-quality data were used in the mapping.

### 2.3. Detection of Single Nucleotide Polymorphisms and Insertion/Deletion Polymorphism

The DNA sequencing reads from *C. trifoliata* and *C. clementina* were aligned to the citrus genome (https://phytozome.jgi.doe.gov/pz/portal.html#!info?alias=Org_Cclementina) [24] separately using Burrows–Wheeler aligner (BWA, http://bio-bwa.sourceforge.net/) software with default parameters [27]. Further, using sequence alignment/map (SAM) tools, the resulting binary alignment/map (BAM) and SAM files were converted for sorting and indexing alignments [27]. ‘Mark duplicates’ in Picard tools (V1.106) was used to discard duplicates, and the final sorted BAM results were used for downstream analysis. To increase the accuracy of the mapped reads, the genome analysis Tookit (GATK, version 1.5; http://www.broadinstitute.org) was used to realign reads [28]. Finally, the GATK was used to detect the single nucleotide polymorphism (SNP) and insertion/deletion polymorphism (InDel), which were filtered with the following criteria below: (1) the coverage of SNPs were between half and twice the average coverage of mapped reads; (2) the quality scores (QUAL) were below 30; (3) there are no more than three SNPs within a 10 nucleotide (nt) region. The average sequencing depth and coverage were calculated using the alignment results [27].

### 2.4. Annotation of Single Nucleotide Polymorphisms and Insertion/Deletion Polymorphism

From the variant call format (VCF) files obtained through filtering, SNPs and InDels were annotated based on genomic location and classified by the likely effects of the variations, including functional categories using SnpEff [29]. The SnpEff binary database file (.bin) was generated using the citrus genome annotation file (gff3) and the genome sequence [24]. This generated database was used to annotate the effects of SNP by region, effect (high, moderate, low, and modifier), and functional class (missense, nonsense, and silent) for all of the individuals. The localization of SNP and InDel was based on the annotation of the gene models of the citrus reference genome [24]. The two types of polymorphisms in the gene region and other genome regions were annotated as genic and intergenic, respectively. The genic SNP and InDel were classified as coding sequences (CDS), untranslated region (UTR), and intron, according to their localization. The SNP in the CDS were further separated into synonymous and non-synonymous amino substitution using SnpEff version [29]. The distribution of SNPs and InDels on each cucumber chromosome was visualized using Circos (http://circos.ca/) [30].

### 2.5. RNA Sequencing Analyses

Total RNA was extracted using TRIzol reagent (Invitrogen Life Technologies, Gaithersburg, MD, USA), and then treated with RNase-free DNase I, according to the manufacturer’s instructions. The total RNA was sent to Beijing Genomics Institute (Shenzhen, China), where the libraries were produced and sequenced using Illumina’s Genome Analyzer (Solexa). Raw sequence reads were filtered for quality using the FASTX-Toolkit (http://hannonlab.cshl.edu/fastx_toolkit/) [31] with default parameters by removing low-quality reads and adaptor sequences. RNA-Seq reads from individual rootstocks, and scions of various graft unions, were separately aligned to the citrus genome [24] using STAR software (http://www.stargroup.uwaterloo.ca/) [32]. Differential expression analysis was performs using the NOIseq package [33]. In this study, a probability ≥0.8 and an absolute value of log2 fold change ≥1 were used as the threshold to judge the significance of the gene expression difference [34]. Gene annotation was conducted using the Blast2GO program [35]. The data from this study have been submitted to the sequence read archive (SRA) under accession number SRP155584.

### 2.6. Detection of Mobile Genes between Scions and Rootstocks from Grafted Plants

To detected SNPs in RNA-seq, we merged the three biological replicates to increase the coverage of RNA-seq. Following alignment, the coverage of each genomic position by base A, G, C, and T was calculated based on the mpileup file generated by SAMtools [36]. Only loci that were homozygous and showed different genotypes between the scions and the rootstocks of a grafted plant were used for downstream transmitting locus identification. The InDel was not considered for detected mobile transcripts. For each homozygous locus, we required at least five reads, supporting the dominant allele in all of the samples, in which the frequency of the dominant allele was greater than 95% [37,38]. A transcript was defined as mobile if its corresponding RNA-Seq reads from the donor were detected in the receptor’s RNA-Seq library. These reads should be perfectly aligned to the donor genome and valid alleles at a SNP site with more than 5% of all of the reads mapping to support it [37,38]. The supported SNP was required to show exactly two different alleles, which had to be consistent and conflict-free across in homograft and heterograft datasets (Supplementary Figure S2). A gene that produces mobile transcripts between scions and rootstocks was described thereafter as a graft-transmitting gene, as in previous studies [37,38].

### 2.7. Real-Time Polymerase Chain Reaction

The differentially expressed genes were confirmed by real-time with SYBR green I (SG) chemistry (QIAGEN, Duesseldorf, Germany), as described previously [39]. The primers were designed with the Primer Express software (PE Applied Biosystems, Foster City, CA, USA) and tested to ensure the amplification of single discrete bands with no primer dimers (Supplementary Table S1). Data was evaluated by calibrator-normalized relative quantification with efficiency correction by using the LightCycler™ 480 software version 1.5 (Roche Applied Science, Mannheim, Germany) and normalized to the expression of *β-actin*. Three biologic repeats were assayed in this study.

## 3. Results

### 3.1. Genome Resequencing, Detection, and Characteristics of Genetic Variations

To identify the genome-wide genetic variation between *C. trifoliata* and *C. clementina*, two resequencing libraries from the two genotypes of citrus were constructed. A total of 121 and 125 million reads were generated for *C. trifoliata* and *C. clementina*, respectively. In *C. trifoliata*, 110.4 million (90.94%) reads were mapped to the reference genome, representing 84.40% of the citrus reference genome. In *C. clementina*, 118 million (94.36%) reads were mapped to the reference genome, representing 97.57% of the citrus reference genome, and indicating that the generated dataset was highly relevant with the reference genome.

**Figure 1:**
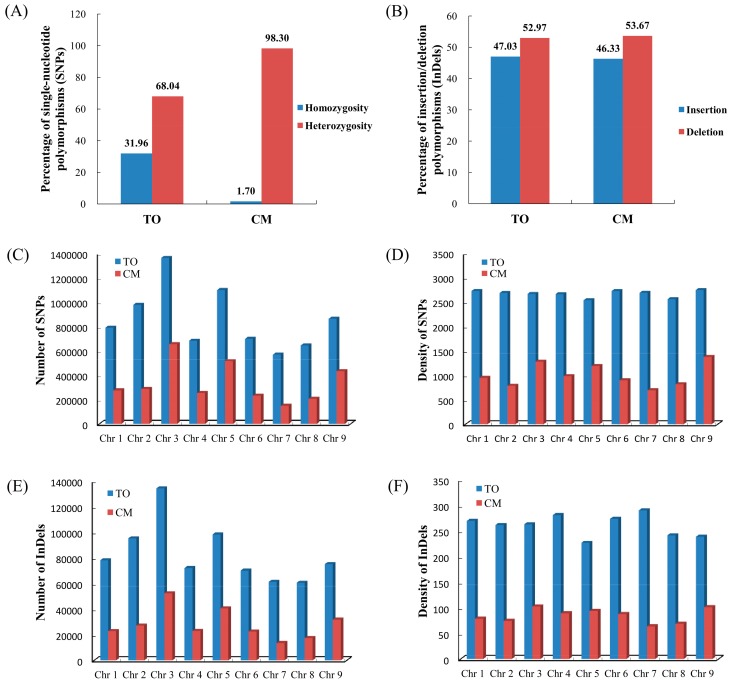
Genetic variation from genome-level distribution of *Citrus trifoliata* and *Citrus clementina*. (**A**) Percentage of homozygous and heterozygous single-nucleotide polymorphisms (SNPs) in *C. trifoliata* (CT) and *C. clementina* (CC); (**B**) Percentage of insertions and deletions of insertion/deletion polymorphisms (InDels) in CT and CC; (**C**,**E**) Number of SNPs and InDels detected on each citrus chromosome; (**D**,**F**) Density of SNPs and InDels detected on each citrus chromosome.

The genome-wide SNPs and InDels of *C. trifoliata* and *C. clementina* were identified by comparing with the reference sequence, respectively. After combining the data, a total of 7,744,203 and 3,062,798 SNPs were detected in *C. trifoliata* and *C. clementina* compared to the citrus reference genome, respectively. The detected SNPs from two citrus species were classified into two groups on the basis of nucleotide substitutions: transitions (A/G and C/T) and transversions (A/C, A/T, C/G, and G/T). In *C. trifoliata*, the identified SNPs comprised 8,243,171 transitions and 4,770,130 transversions. Among these detected SNPs, 2,475,105 (31.96%) were heterozygous, and 5,269,098 (68.04%) were homozygous (Figure 1A). The obtained SNPs from *C. clementina* included 2,019,022 transitions and 1,095,896 transversions. Approximately 3,010,678 (98.30%) were heterozygous, and only 52,120 (1.70%) were homozygous (Figure 1A). Considering SNP density, 26,708 SNPs/Mb for *C. trifoliata* and 10,562 SNPs/Mb for *C. clementina* were discovered. In addition, the transition/transversion ratio was observed, with an average of 1.73 and 1.84 for *C. trifoliata* and *C. clementina*, respectively. Furthermore, a total of 747,897 and 252,679 InDels were identified in *C. trifoliata* and *C. clementina*, respectively. Among these detected InDels, *C. trifoliata* had 350,985 (47.03%) insertions and 396,912 (52.97%) deletions; *C. clementina* had 115,624 (46.33%) insertions and 137,055 (53.67%) deletions (Figure 1B). The approximate InDel density variant rate that was observed for each genotype genome was one change in every 393 bases (*C. trifoliata*) and 1174 bases (*C. clementina*).

The citrus reference genome, including scaffolds one to nine (equivalent to nine citrus chromosomes), was used for analyzing the chromosomal distributions of the identified SNPs and InDels (Supplementary Table S2). The SNPs and InDels of *C. trifoliata* and *C. clementina* were widely distributed to each chromosome. However, there was a uniform distribution of SNPs and InDels in the nine chromosomes of both citrus species (Supplementary Figure S3). A total of 7,684,524 SNPs from *C. trifoliata* were identified throughout nine chromosomes. Chromosome 3 (chr 3) showed the highest number of SNPs (1,362,505), and very few SNPs (568,682) were detected on chromosome 7 (Figure 1C). The density of SNPs in each chromosome varied from 2539 SNP/100kb (chr 5) to 2746 SNP/100kb (chr 9). Similarly, chromosome 3 showed the highest number of SNPs (653,335), and very few SNPs (147,176) were detected on chromosome 7 in *C. clementina* (Figure 1D). The frequency of SNPs in each chromosome varied from 696 SNP/100kb (chr 7) to 1377 SNP/100kb (chr 9). The length of InDels ranged between one bp to 40 bp in the two citrus species. In this study, the largest InDel size (40 bp) was detected in *C. clementina*, and most of the InDels were less than 10 bp. Further, a high rate of single-nucleotide InDels existed in the two citrus species. Compared to the reference genome, the highest abundance of InDels of *C. trifoliata* was detected in chr 3, and a small proportion of InDels was detected in chr 8. The remaining chromosomes showed similar patterns of InDel distribution (Figure 1E). For *C. clementina*, the highest abundance of InDels was detected in chr 3, and a small proportion of InDels was detected in chr 7 (Figure 1F). The remaining chromosomes also showed similar patterns of InDel distribution. The short InDels (one to five base pairs) may have deleterious effects on the functionality of genes during expression or transcriptional processes.

### 3.2. Analysis of Genetic Variation between C. Trifoliata and C. Clementina

**Figure 2:**
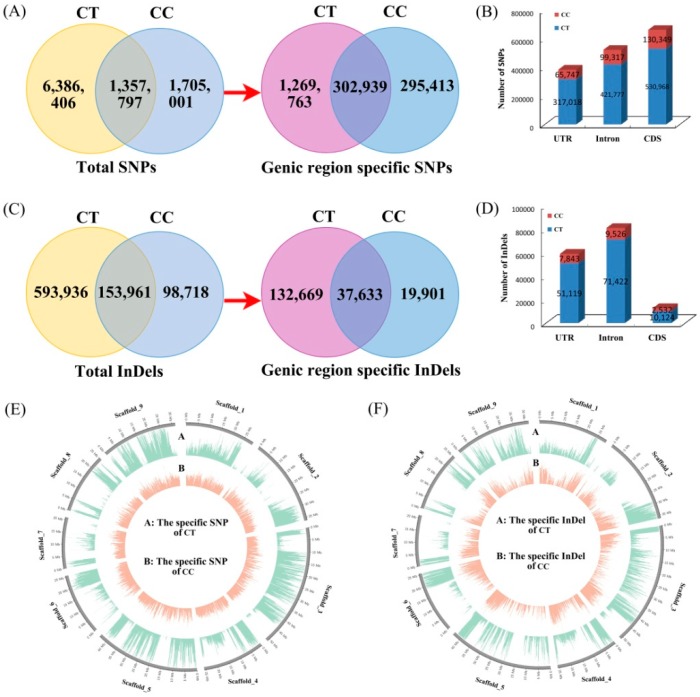
Venn diagrams of SNPs and InDels in *C. trifoliata* (CT) and *C. clementina* (CC). (**A**) Venn diagrams of total SNPs and specific SNPs; (**B**) Distribution of SNPs in genic; (**C**) Venn diagrams of total InDels and specific InDels; (**D**) Distribution of InDels in genic; (**E**,**F**) Landscape of the genome variation of CT versus CC.

To identify the specific genetic variation between *C. trifoliata* and *C. clementina*, we compared the SNPs and InDels from two citrus species. A total of 9,449,204 SNPs were identified in the two citrus species, of which 1,357,797 were identified to be common, and 8,091,407 (6,386,406 and 1,705,001 SNPs in *C. trifoliata* and *C. clementina*, respectively) were specific in the two genotypes (Figure 2A). To further evaluate the differential SNPs in the genic region, a total of 1,868,115 (19.77%) SNPs from the two citrus species were located in the genic region. Among these genic SNPs, 302,939 were found in both *C. trifoliata* and *C. clementina*, while 1,269,763 were specific to *C. trifoliata* and distributed in 21,385 genes, and 295,413 were specific to *C. clementina* and distributed in 19,395 genes (Figure 2A). The positions of specific SNPs were identified in CDS, intron, and UTR regions, according to the reference genome (Figure 2B). Among the 1,565,176 specific SNPs, 382,765 (24.46%) were located in UTR regions (*C. trifoliata*: 317,018 and *C. clementina*: 65,747), 521,094 (33.30%) were located in intron regions (*C. trifoliata*: 421,777 and *C. clementina*: 99,317), and 661,317 (42.24%) were located in CDS regions (*C. trifoliata*: 530,968 and *C. clementina*: 130,349). The number of non-synonymous SNPs was 280,502 (52.83%) and 71,705 (55.01%) in CDS regions of *C. trifoliata* and *C. clementina*, respectively. Meanwhile, 5636 (1.06%) and 1656 (1.27%) other SNPs gave rise to variants in the start or stop codons of coding genes in *C. trifoliata* and *C. clementina*, respectively (Supplementary Table S3).

A total of 846,615 InDels were identified between *C. trifoliata* and *C. clementina*, of which 153,961 were common, and 692,654 (593,936 and 98,718 SNPs in *C. trifoliata* and *C. clementina*, respectively) were specific in the two citrus species (Figure 2C). After being carefully filtered, 190,199 (22.47%) InDels were obtained in the genic region, of which 37,633 (4.45%) were found in both citrus species (distributed in 12,109 genes), 132,665 (15.67%) were specific to *C. trifoliata* and distributed in 19,679 genes, and 19,901 (2.35%) were specific to *C. clementina* and distributed in 9250 genes (Figure 2C). Among the 152,566 specific genic InDels from the two citrus species, 58,962 (38.65%) were located in UTR regions (*C. trifoliata*: 51,119 and *C. clementina*: 7843), 80,948 (53.06%) were located in intron regions (*C. trifoliata*: 71,422 and *C. clementina*: 9526), and 12,656 (8.29%) were located in CDS regions (*C. trifoliata*: 10,124 and *C. clementina*: 2532). Within the gene body, the maximum number of specific SNPs (*C. trifoliata*: 41.82% and *C. clementina*: 44.12%), and only a small proportion of specific InDels (*C. trifoliata*: 7.63% and *C. clementina*: 12.72%) from genic regions were present in the CDS regions (Figure 2D). The lengths of the InDels and their frequency in *C. trifoliata* and *C. clementina* were calculated. Among the 846,615 InDels, 353,581 insertions (one to five bp), and 366,991 deletions (one to five bp) were observed. The frequency of different types of InDels varied, and was negatively correlated with the number of nucleotides. Mononucleotide InDels (453,154, 53.53%) were the most frequent InDel in *C. trifoliata* and *C. clementina*, following by dinucleotide (122,708, 14.49%) and trinucleotide InDels (69,124, 8.16%). The specific SNPs and InDels from *C. trifoliata* and *C. clementina* were also visualized in the citrus reference chromosome using Circos (Figure 2E,F). An uneven distribution of SNPs and InDels was observed between the short and long chromosome arms of the citrus.

### 3.3. Annotation of Species-Specific Single Nucleotide Polymorphisms and Insertion/Deletion Polymorphism

**Figure 3:**
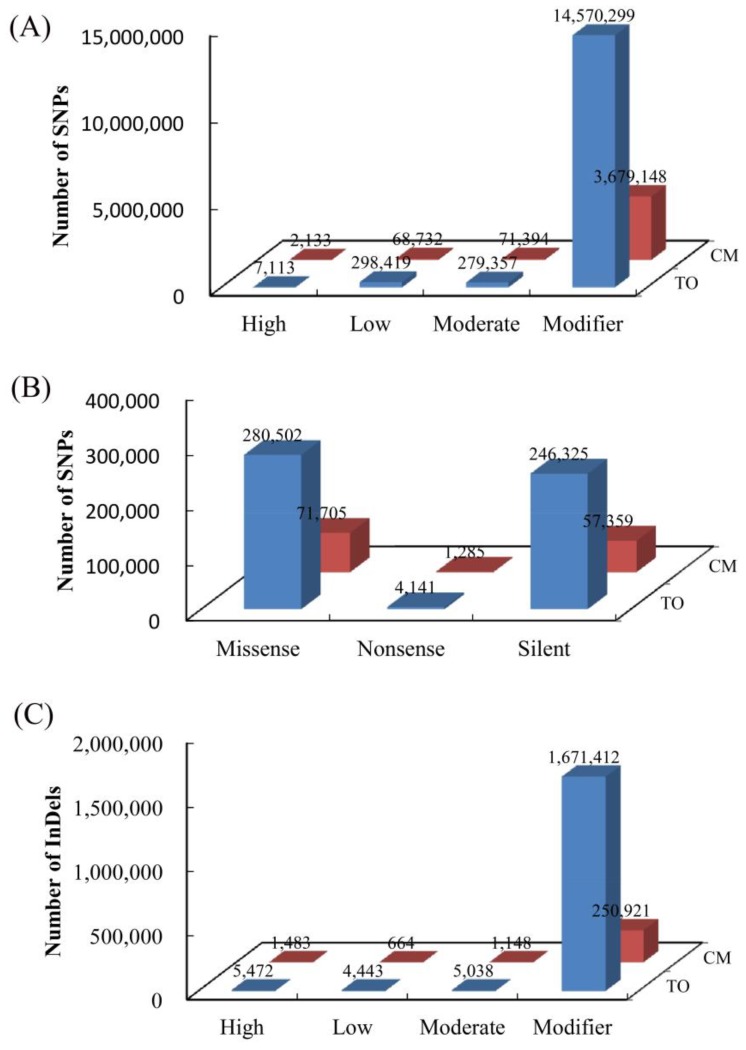
Impact categories of specific genetic variation from CT and CC. (**A**) Impact categories of specific SNPs; (**B**) Functional classes of the detected specific SNPs; (**C**) Impact categories of specific InDels.

To annotate the detected specific SNPs and InDels between *C. trifoliata* and *C. clementina*, sequence information and annotation files of the citrus genome were used to evaluate the possible effects of the variants in genomic, exonic, and functional categories. In all, 14,570,229 (98.12%) and 3,679,148 (98.10%) SNPs were classified as sequence modifiers (introns or affecting non-coding genes), and a moderate effect accounted for 279,357 (1.88%) and 71,394 (1.90%) of the SNP in *C. trifoliata* and *C. clementina*, respectively (Figure 3A). The low-effect variants were 298,419 (1.97%) and 68,732 (1.80%) in *C. trifoliata* and *C. clementina*, respectively. The remaining high-effect variants were 7113 (0.05%) and 2133 (0.06%) in *C. trifoliata* and *C. clementina*, respectively (Figure 3A). Functional classes of missense, nonsense, and silent variants were evaluated for the detected specific SNPs. A total of 4141 SNPs (0.78%) were considered as high-effect variants (nonsense mutation) in *C. trifoliata*, which included 280,502 (52.84%) missense SNPs and 246,325 (46.37%) silent SNPs, with a 1.14 missense/silent ratio. In *C. clementina*, 1285 SNPs (0.99%) were assigned as high effect and comprised 71,705 (55.01%) missense SNPs and 57,359 (44.01%) silent SNPs, with a missense/silent ratio of 1.25 (Figure 3B). A similar methodology was used to annotate the detected specific InDel. Approximately 1,671,412 (99.11%) and 250,921 (98.70%) InDels were classified as sequence modifiers, and 5038 (0.30%) and 1148 (0.45%) were classified as moderate effect in *C. trifoliata* and *C. clementina*, respectively (Figure 3C). The percentages of low-effect InDels were fairly small in *C. trifoliata* 4443 (0.26%) and *C. clementina* 664 (0.26%). The remaining high-effect variants accounted for 5472 (0.32%) and 1483 (0.58%) in total in *C. trifoliata* and *C. clementina*, respectively (Figure 3C).

To confirm the reliability of these genetic variations, 30 primer pairs (including 60 SNPs and eight InDels) were selected randomly and verified in two citrus species (Supplementary Table S1). Of these primer pairs, two could not amplify any fragment, suggesting that these primers were not well-designed; five amplified numerous non-target bands, suggesting that these primers were also problematic, and 25 primer pairs were successfully amplified. Twenty (including 26 homozygous SNPs, six heterozygous SNPs, and six homozygous InDels) of the successfully amplified primer pairs were validated by Sanger sequencing (Supplementary Table S4), and five did not exhibit any difference between the two citrus species. These results suggested that genome resequencing was capable of successfully identifying genetic variation from the two citrus species.

### 3.4. Messenger RNA Movement between Citrus Trifoliata and Citrus Clementina

To detect the gene exchange between scions and rootstocks, we devised a strategy based on grafting two different citrus species displaying a high frequency of genomic sequence SNPs. The species-specific SNPs that were present in long RNA molecules allowed us to identify their origin—and thus, their mobility—in grafted plants (Supplementary Figure S2). Two sets of grafted materials, one from the leaves of grafted plants and the other from the stems of grafted plants, were investigated in this study. About 32 to 46 million reads were produced for each individual RNA-Seq library, and about 90% of these reads were mapped to the citrus reference genome. We mapped genomic sequencing reads of the scions and rootstocks to the citrus reference genome, determined their genotypes, and identified the diagnostic SNP loci between the respective scions and rootstocks.

**Figure 4:**
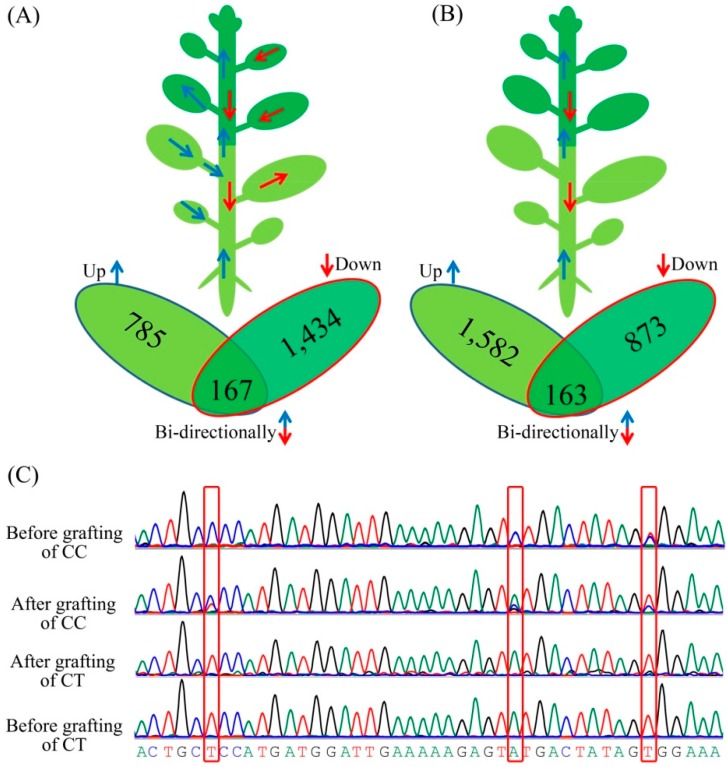
Diagrams of messenger RNA (mRNA) movement in CT and CC. (**A**) mRNA movement in the leaves between CT and CC; (**B**) mRNA movement in the stems between CT and CC. Up and down arrows and their pointing numbers respectively represent the moving directions and numbers of genes producing mRNAs moved into scions (up) or rootstocks (down); (**C**) The transmitting genes as confirmed by Sanger sequencing.

The number of transmitting genes varied among the different grafted materials. For example, a total of 2386 transmitting genes were detected in the leaves between *C. trifoliata* and *C. clementina* (Supplementary Table S5), 1601 transmitting genes were found to be transferred from the scions to the rootstocks, and 952 transmitting genes were found to be transferred from the rootstocks to the scions (Figure 4A). Between these two sets of transmitting genes from the leaves, 167 were transmitted bidirectionally into both scions and rootstocks. In contrast, 2618 transmitting genes were detected in the stem of *C. trifoliata* and *C. clementina* (Supplementary Table S6), 1036 transmitting genes were found to be transferred from the scions to the rootstocks, and 1745 transmitting genes were found to be transferred from the rootstocks to the scions (Figure 4B). Between these two sets of transmitting genes from the stems, 163 were transmitted bidirectionally into both scions and rootstocks. Among them, 608 transmitting genes were detected in two different graft materials. Collectively, 4396 transmitting genes were identified from these two sets of grafted materials. To investigate the mobility of the identified transcripts further, Sanger sequencing (Figure 4C) was conducted on 63 transmitting genes. However, only 11 were confirmed in this study (Supplementary Table S7).

### 3.5. Classification of the Biological Functions of the Transmitting Genes

**Figure 5:**
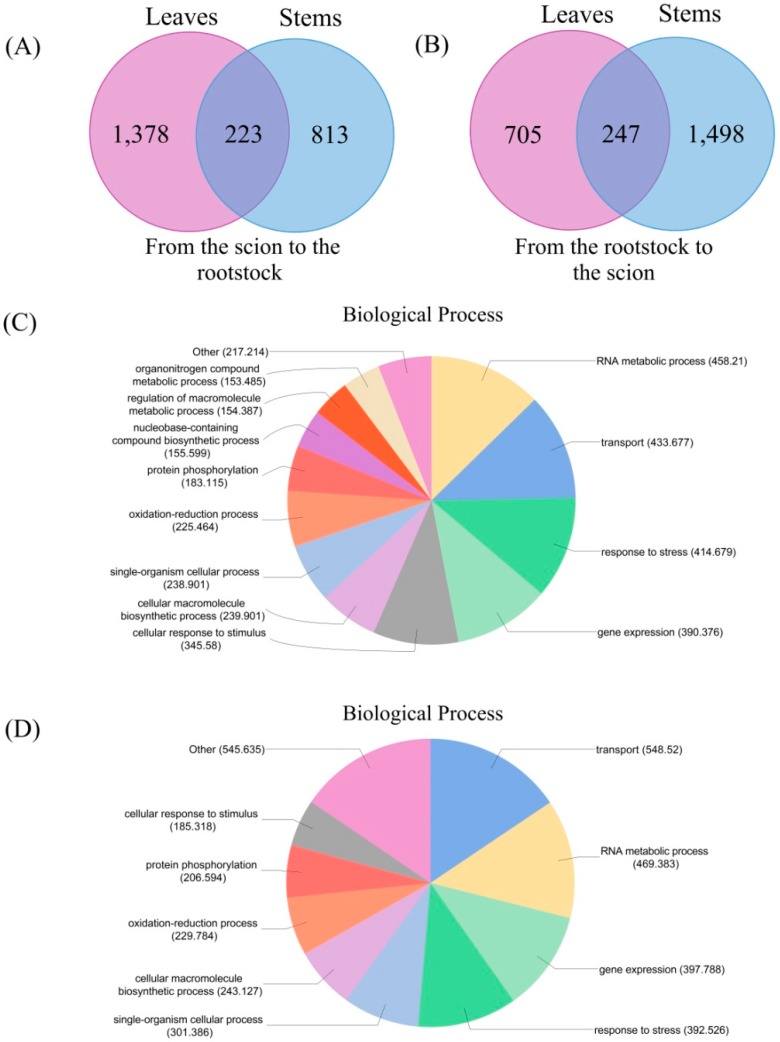
Analysis of transmitting genes. (**A**) Venn diagrams of leaves and stems transmitting genes from scions and rootstocks; (**B**) Venn diagrams of leaves and stems transmitting genes from rootstocks and scions; (**C**) Gene Ontology (GO) annotation transmitting genes from scions and rootstocks; (**D**) Gene Ontology (GO) annotation transmitting genes from rootstocks and scions.

Our experiments revealed specificity, in terms of transcript delivery through the xylem and phloem, to the leaves and stems. For example, a total of 2450 transmitting genes were found to be transferred from the scions to the rootstocks when the data from the leaves and stems were combined, while only 247 genes were held in common (Figure 5A). Furthermore, 2414 transmitting genes were found to be transferred from the rootstocks to the scions. Among them, 223 transmitted genes were overlapped between the leaves and stems (Figure 5B). These findings support the hypothesis that a molecular mechanism functions, at the vascular tissue level, to impart specificity in the targeting of the xylem and phloem mobile genes to certain sinks. The transmitting genes from the scions to the rootstocks were associated with a range of processes. Consistent with the roles played by hormones in local and developmental reprogramming, a significant number of the identified transmitted genes were found to be associated with hormone-metabolic and hormone-mediated signaling. Many kinases, cell wall organization, and cell cycle-regulated genes were also found. The transmitting genes from the rootstocks to the scions were associated with a range of genes, including responses to certain forms of stresses or stimuli, photosynthesis, signal transduction, and some transcription factors. These genes provided additional evidence to support the hypothesis that mRNA exchange during grafting process was extensive and on a genome-wide scale.

The functional characteristics of transmitting genes still remained largely unknown, and analysis of their biological functions by gene ontology (GO) term analysis may provide valuable hints regarding their functions during the grafting process. For the categories based on biological processes, a GO term analysis of the 2450 transmitting genes from the scions to the rootstocks (Figure 5C) and 2414 transmitting genes from the rootstocks to the scions (Figure 5D) revealed that these transmitting genes were involved in 12 and nine main biological processes in the rootstocks and scions, respectively. The three largest groups were RNA metabolic processes, transport, and response to stress. A comparative analysis of the biological processes indicated that the organonitrogen compound metabolic process, regulation of the macromolecule metabolic process, and nucleobase-containing compound biosynthetic process were enriched in scions, and cellular response to stimulus and protein phosphorylation were enriched in rootstocks. The biological interpretation of these transmitting genes was further investigated by Kyoto encyclopedia of genes and genomes (KEGG) pathway analysis. In *C. trifoliata* and *C. clementina*, 31 and 23 different pathways were found, respectively, of which some were consistent with GO analysis. Those pathways were mainly correlated with the development that is involved with metabolism, hormone signal transduction, and transcriptional regulation. We further analyzed the 334 genes whose mRNAs moved bidirectionally in the graft scion of leaves and stems (Supplementary Table S6). Nine overrepresented biological processes were identified, including defense response, protein phosphorylation, and other forms of abiotic stresses and processes related to translation elongation and photosynthesis.

### 3.6. Identification of Differentially Expressed Genes (DEGs) during Grafting Process

**Figure 6:**
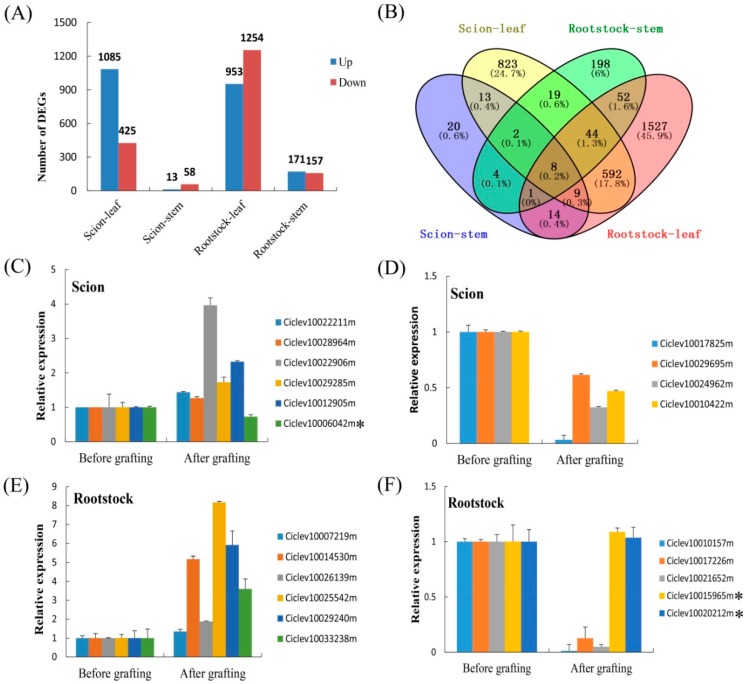
Analysis of differential expression of genes. (**A**) The total numbers of differentially expressed genes between scions and rootstocks; (**B**) Venn diagrams of differentially expressed genes from different tissues of scions and rootstocks. (**C**) Real-time polymerase chain reaction (PCR) confirmation of the differentially expressed genes (DEGs) in scion. (**E**,**F**) Real-time polymerase chain reaction (PCR) confirmation of the differentially expressed genes (DEGs) in rootstock. The asterisk indicates that these genes showed different expression patterns between real-time PCR and RNA sequencing. Relative transcript levels are calculated by real-time PCR with *Actin* as a standard. Data are means ±standard error (SE) of three separate measurements.

To analyze the transcriptome changes of scions and rootstocks during the grafting process, RNA-seq was performed on the stems and leaves after grafting and before grafting. In this study, 1510 genes were DEGs after the grafting of leaves compared with the before-grafting leaves, of which 1085 genes were up-regulated and 425 were down-regulated based on a probability ≥0.8 and an absolute log2 fold change value ≥1 (Figure 6A). Meanwhile, 71 genes were DEGs in after-grafting stems compared with before-grafting stems, of which 13 genes were up-regulated and 58 were down-regulated in scions (Supplementary Table S8). In rootstocks, 2247 genes were DEGs in leaves, of which 953 genes were up-regulated and 1294 were down-regulated; while 328 genes were DEGs in stems, of which 171 genes were up-regulated and 157 were down-regulated (Supplementary Table S8). By combining the results from the scions, a total of 1549 DEGs genes were identified, and 32 were shared among stems and leaves (Figure 6A). When the data from the rootstocks were combined, a total of 2470 non-redundant DEGs was identified, while 105 were shared among the stems and leaves (Figure 6B). To validate the reliability of the expression patterns obtained from RNA-Seq, 21 randomly selected DEGs were investigated by real-time PCR. Although the exact fold change for the selected genes varied between RNA-seq and real-time PCR analysis, the gene expression trend of the two approaches was largely consistent (Figure 6C–F). This indicates that the RNA-Seq data that are reported here are valuable.

We performed GO term analysis on the 1549 and 2470 DEGs from scions and rootstocks, respectively. The analysis revealed that these DEGs were involved in 12 and seven main biological processes in scions and rootstocks, respectively. The three largest groups were the single-organism process, oxidation–reduction process, and carbohydrate metabolic process. A comparative analysis of the biological processes from scions and rootstocks indicated that the cell wall organization or biogenesis, response to oxidative stress, and movement of cell or subcellular components were enriched in scions, and photosynthesis was enriched in rootstocks. An overview of biotic stresses was performed with MapMan software to visualize the stress pathways in which DEGs were involved (Supplementary Figure S4). In the comparison of scions and rootstocks, the DEGs were distributed among all of the stress pathways, and mainly concentrated in cell wall, signaling, and secondary metabolism, with several genes belonging to the redox state, peroxidases, and heat shock proteins. Moreover, several DEGs were involved in hormonal signaling cascades, particularly in the activity of transcription factors such as basic region/leucine zipper motif (bZIP), WRKY, and MYB. It is worth noting that the DEGs from the scions that were involved in these pathways were up-regulated (Supplementary Figure S4A), while rootstocks and the scions were the opposite (Supplementary Figure S4B). Overall, these pathway changes affect plant responses to abiotic/biotic stresses.

## 4. Discussion

Gene-based molecular markers are increasingly used in crop-breeding programs for marker-assisted selection. However, the identification of genetic variants that are associated with important agronomic traits has remained a difficult task in citrus [24]. One of our main objectives was to identify the specific genetic variation between *C. trifoliata* and *C. clementina*. Genome resequencing is an effective strategy to discover a large number of genetic variants in plant genomes. Therefore, genome analysis based on resequencing these two citrus species was used for the comprehensive identification of SNPs and InDels. A total of 9,449,204 SNPs and 846,616 InDels were identified between *C. trifoliata* and *C. clementina*. These detected genetic variants from this study will expand the genomic resources that are available for citrus, and can be used to develop genotyping platforms to perform a marker–trait association study. In addition, we identified and characterized the patterns of genome-wide gene exchanges across graft junctions in grafted citrus using diagnostic SNPs derived from species-specific SNPs. The large genomic-scale exchanges of genes between scions and rootstocks could be the key genetic basis of the superior performance of grafted plants.

There has been concerted interest in the last few years in the analysis of genetic variation in some important horticultural plants [40,41]. Furthermore, studies of genetic variants offer a potentially large and untapped reservoir of genetic variation in heterogeneous genetic backgrounds that can be exploited in studies of gene function, evolution, and targeted selection experiments [40,42]. Since the completion of the whole genome sequence of citrus and the development of technologies to detect SNPs, several different reports on polymorphism identification have been published [24,43]. For example, genomic analyses of primitive, wild, and cultivated citrus have provided new insights into citrus apomixis, and constitute a promising resource for the mining of agriculturally important genes by using genetic, genomic, and transcriptomic approaches [40]. Recently, the segmental ancestry of 46 citrus accessions was delineated using 588,583 ancestry-informative SNPs derived from three citrus species [42]. In this study, we identified large numbers of genetic variants between *C. clementina* and *C. trifoliata*. Interestingly, the number of specific genetic variations (including SNPs and InDels) from *C. clementina* was less than that of *C. trifoliata* in this study, which indicates that *C. clementina* is more closely related to the reference genome than *C. trifoliata*. Among the SNPs of the CDS regions, the frequency of non-synonymous substitutions was much higher than that of synonymous substitutions. Similar mutagenic effects were also observed in other citrus species [43]. Non-synonymous changes may contribute to phenotypic differences because they alter amino acid sequences [44]. Meanwhile, the InDels and SNPs that occur in the CDS region of important genes could be seen to affect gene function by frameshifts and structural changes of the protein. In this study, annotation analysis showed that approximately 661,317 (7.00% of total) specific SNPs and 12,656 (1.49% of total) specific InDels from two citrus species were located in the CDS region. Meanwhile, the high-effect genetic variations causing stop/start codon changes and frameshifts were also detected in the two citrus species. These genetic variations may represent the causal genetic variation contributing to the phenotype variation between *C. trifoliata* and *C. clementina*. Therefore, the high frequency of non-synonymous changes and frameshift InDels could help explain the differences in agronomic traits between *C. trifoliata* and *C. clementina*.

Successful grafting may improve the quality and production of the horticultural plants by enhancing nutrient absorption and conferring resistance to many biotic and abiotic stresses in the graft units [3]. The long-distance transport of diverse molecules contributes to explain the molecular mechanism for how graft partners interact with each other to produce a successful graft union [45]. Recently, a number of studies have also reported on the ability of transcriptomics assays to identify the graft transmissibility of genes in models, and some woody plants by specific genetic variation markers [38,46]. However, most reports were based on model species, and how the results from these studies can be applied to agricultural graft crops is unknown. Here, a total of 4396 annotated genes were found to produce mobile transcripts across graft junctions. They accounted for about 12.96% of the total protein coding genes (33,929) in citrus. The extent of mRNA exchange between graft partners that was revealed was extensive; it was at a similar scale (about 12.7%) as what was recently reported in grape [38]. Since the detection of mobile genes is contingent on the availability of SNPs differentiating graft partners, growth stages, and growth environments, it would not be possible to detect all of the mobile mRNAs [38]. Therefore, the proportion of the mobile mRNAs is likely underestimated in this study. The transmitting genes were involved in many different biological processes. It was interesting to note that many processes that are related to the responses to various forms of stresses and stimuli were over-represented, suggesting that gene movement was responsive to growth conditions and environmental stresses. In addition, we discovered that there were some biological processes shared between the citrus in this study and the *Arabidopsis* that was previously reported [38].

Comparisons of the abundance, movement directions, and patterns of transmitting genes in the stem and leaves revealed an important fact: while thousands of genes could transmit their mRNAs between scions and rootstocks, only a small number of them might reach certain tissues to become biologically relevant (approximately 13.83% of the transmitting genes were shared between the stems and leaves). For example, a high number of mRNAs were found to be transported from *Arabidopsis* into the parasitic Cuscuta in the Cuscuta–*Arabidopsis* study [46]. However, as the actual distance between the donor and recipient tissues that was analyzed was short, the possibility of local cell-to-cell movement for mRNAs cannot be discounted [46,47]. Based on previous studies, transcription factors that are involved in development and hormone signaling are among the genes whose mRNAs were often found in plant phloem samples; some of which were confirmed were grafting-transmissible, including NAC-domain containing proteins (NACPs), BEL-like homeodomain (BELH), KNOTTED1-like homeobox genes ((KNOX1)), GA-insensitive RNA, and a few auxin/indole-3-acetic acid genes [16,19,48]. In this study, mRNAs for many of these genes were also found to be mobile. Furthermore, mobile genes were detected for many genes encoding proteins involved in the metabolic and signaling pathways of different plant hormones, including auxin, gibberellin, abscisic acid, ethylene, and jasmonic acid [20,46]. These results not only confirmed some of the previous observations, but also provided further evidence that these categories of genes in general were more likely to produce mobile mRNAs in grafted plants. However, further efforts will be made to find more direct evidence, because this was a preliminary, inconclusive deduction on our part.

A larger signaling molecules exchange between scions and rootstocks through the graft union, and small differences in signal concentration or in their receptors/targets, can alter gene expression. Therefore, we analyzed the transcriptomes of the leaves and stems of grafting plants during the grafting process. A total of 1549 and 2470 DEGs were identified in scions and rootstocks, respectively. For the categories based on biological processes, the analysis revealed that these DEGs were associated with the oxidation-reduction process, carbohydrate metabolic process, and abiotic stimulus responses. He et al. [23] studied the (in)compatibility reactions between ‘Hongmian miyou’ (*Citrus grandis* L. Osbeck) and (*C. trifoliata* and fragrant citrus) rootstocks, and a total of 1950 DEGs were identified. The KEGG pathway enrichment analysis revealed that these genes were involved in carbohydrate metabolism, energy metabolism, amino acid metabolism, and plant hormone signal transduction. Liu et al. [22] performed a comparative analysis of ‘Shatangju’ mandarin grafted onto five rootstocks, and the results showed that more DEGs were involved in oxidoreductase function and hormonal signal transduction, and the glycolytic pathways were enriched in ‘Red tangerine versus Canton lemon’. In terms of carbohydrate metabolism, sugars that were involved in priming plant defense responses against fungal pathogens have been reported in rice [49]. Proteins related to abiotic stimulus responses have also been reported to be involved in the grafting process [9,12]. The analysis of gene functional categories revealed that the majority of DEGs are enriched in cell wall organization or biogenesis, which is a response to oxidative stress in scions. Meanwhile, the photosynthesis gene was enriched in rootstocks. Previous study indicated that rootstocks can also affect the growth of scions by nutrient and water uptake, hormonal communication, changes in gene expression, and vice versa [1]. This comprehensive analysis provides fundamental information on the candidate genes and secondary metabolism pathways that are involved in the grafting process for citrus.

## 5. Conclusions

Here, we performed whole genome resequencing to reveal the comprehensive genetic variation between *C. trifoliata* and *C. clementina*. A total of 9,449,204 SNPs and 846,615 InDels were identified in the two citrus species. Meanwhile, a total of 8,091,407 specific SNPs and 692,654 specific InDels were identified in the two citrus genotypes. These genetic variations provided a foundation for the further exploration of citrus diversity and gene–phenotype relationships, and for future research on molecular breeding to improve citrus and related species. Using these diagnostic SNPs derived from high throughput genome sequencing, we identified and characterized the patterns of genome-wide mRNA exchanges across graft junctions between scions and rootstocks. More than 4000 genes transporting mRNAs were identified across graft junctions. These genes were involved in diverse biological processes such as metabolism, hormone signal transduction, and transcriptional regulation. In addition, a total of 3326 DEGs were also found in the scions and the rootstocks after grafting was compared with the control. The results that were obtained in this study further improve our knowledge of the molecular and metabolic changes that are involved in this interaction between the rootstocks and the scions.

## Supplementary Materials

Figure S1. Morphological and cytological analysis of grafted plants. (A). The grafted plants after four months; (B) The middle xylem slitting of grafted plants; (C). Longitudinal diagram of graft union; (D). Crosscutting structure of graft union. Figure S2. Detection of mobile genes. Illustrated are examples for the scions and rootstocks of gene movement detected in this study. Figure S3. Landscape of the genetic variation of *C. clementina* and *C. trifoliata*. Figure S4. MapMan software output to provide an overview of the effect of scions (A) and rootstocks (B) induced on biotic stress responses. Up-regulated and down-regulated transcripts are shown in red and blue, respectively. Table S1. Primers used in this study. Table S2. Number and densities of SNPs and InDels in *C. trifoliata* and *C. clementina* genomes. Table S3. Summary of genetic variations identified from *C. trifoliata* and *C. clementina*. Table S4. Sanger sequencing validated the genetic variations between *C. clementina* and *C. trifoliata*. Table S5. Mobile mRNAs detected in leaves and stems. Table S6. Mobile mRNAs transmitted bi-directionally into both scions and rootstocks. Table S7. Confirmation of the identification of mobile genes scions and rootstocks. Table S8. Analysis of the differentially expressed genes from scions. Table S9. Analysis of the differentially expressed genes from rootstocks.
