# Supplementary material for: Identification of the Genetic Variation and Gene Exchange between Citrus Trifoliata and Citrus Clementina
Source: Biomolecules. 2018 Dec 19;8(4):182. doi: 10.3390/biom8040182 (PMC6315893; doi:10.3390/biom8040182)
Supplement: Supplementary file 1 [file biomolecules-08-00182-s001.zip › Supplementary Figure S1-S4.pdf]

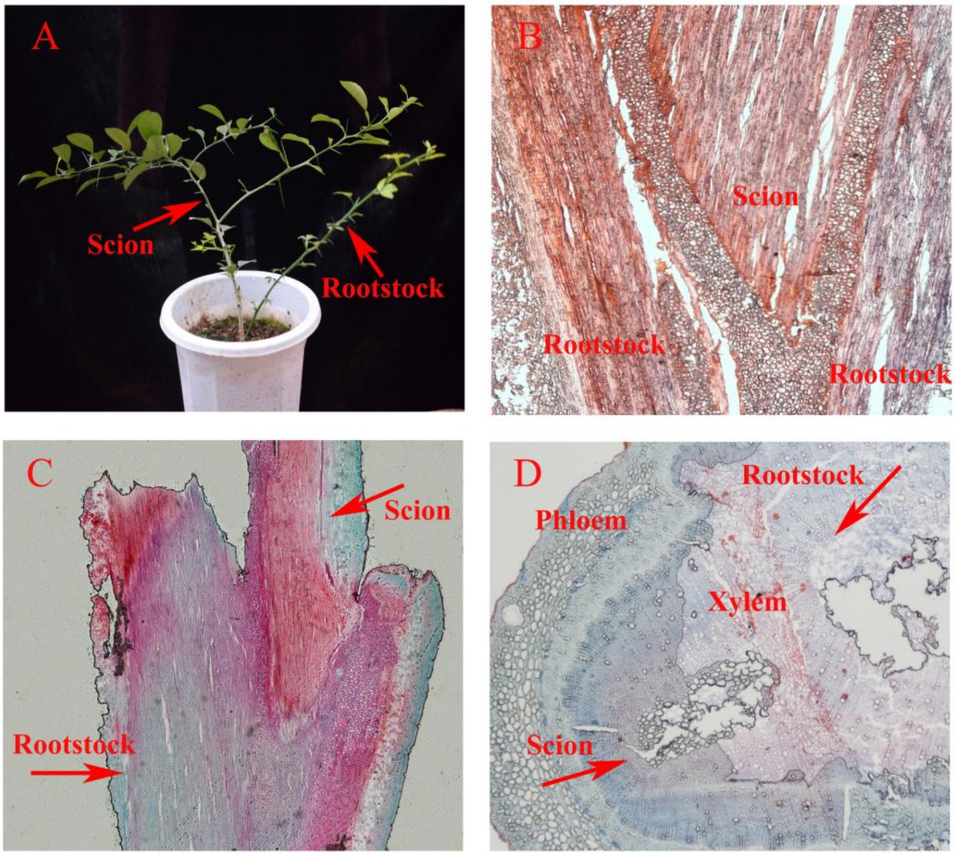

Figure S1. Morphological and cytological analysis of grafted plants. (A). The grafted plants after four months; (B) The middle xylem slitting of grafted plants; (C). Longitudinal diagram of graft union; (D). Crosscutting structure of graft union.

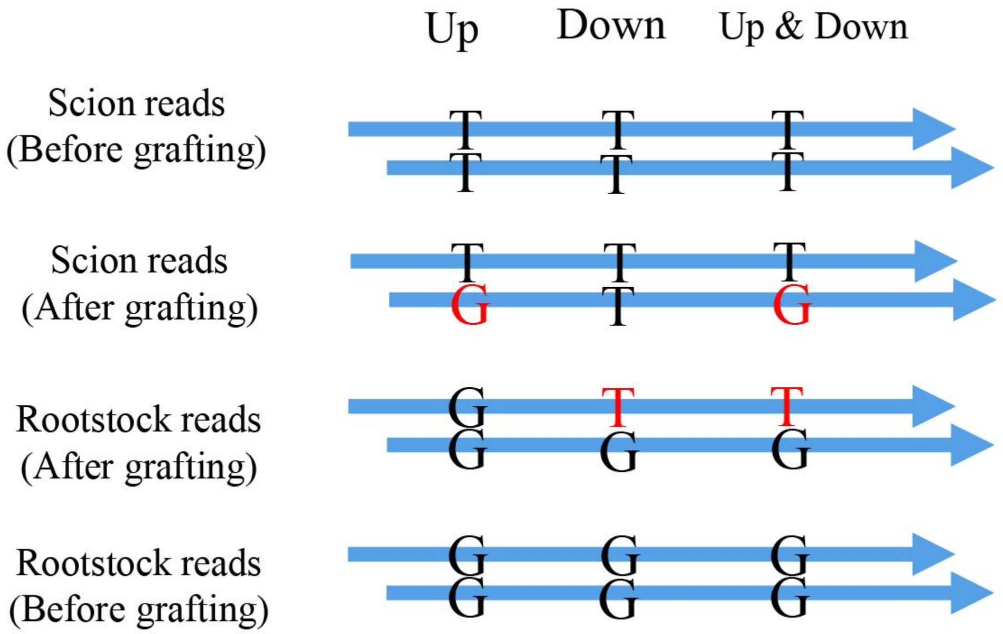

Figure S2. Detection of mobile genes. Illustrated are examples for the scions and rootstocks of gene movement detected in this study.

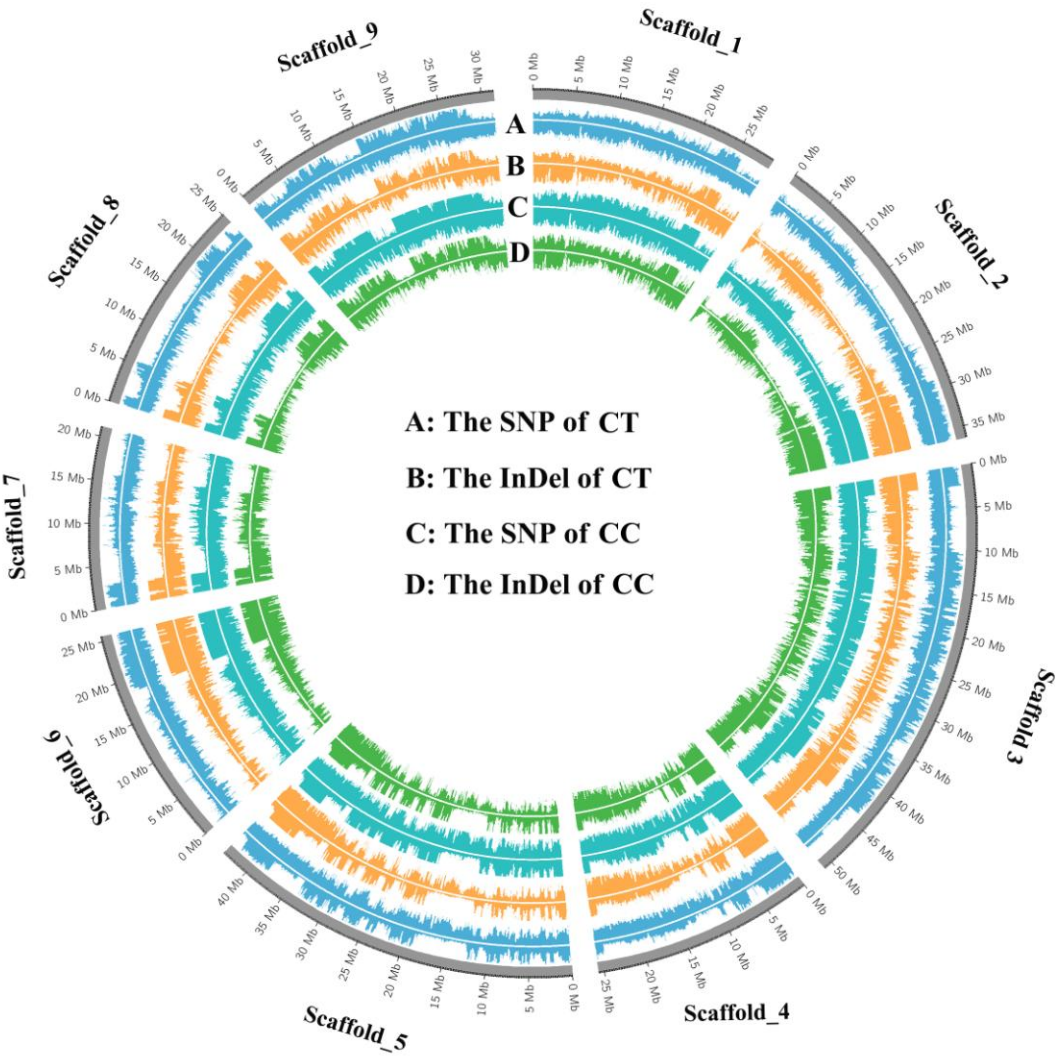

Figure S3. Landscape of the genetic variation of *C. clementina* and *C. trifoliata*.

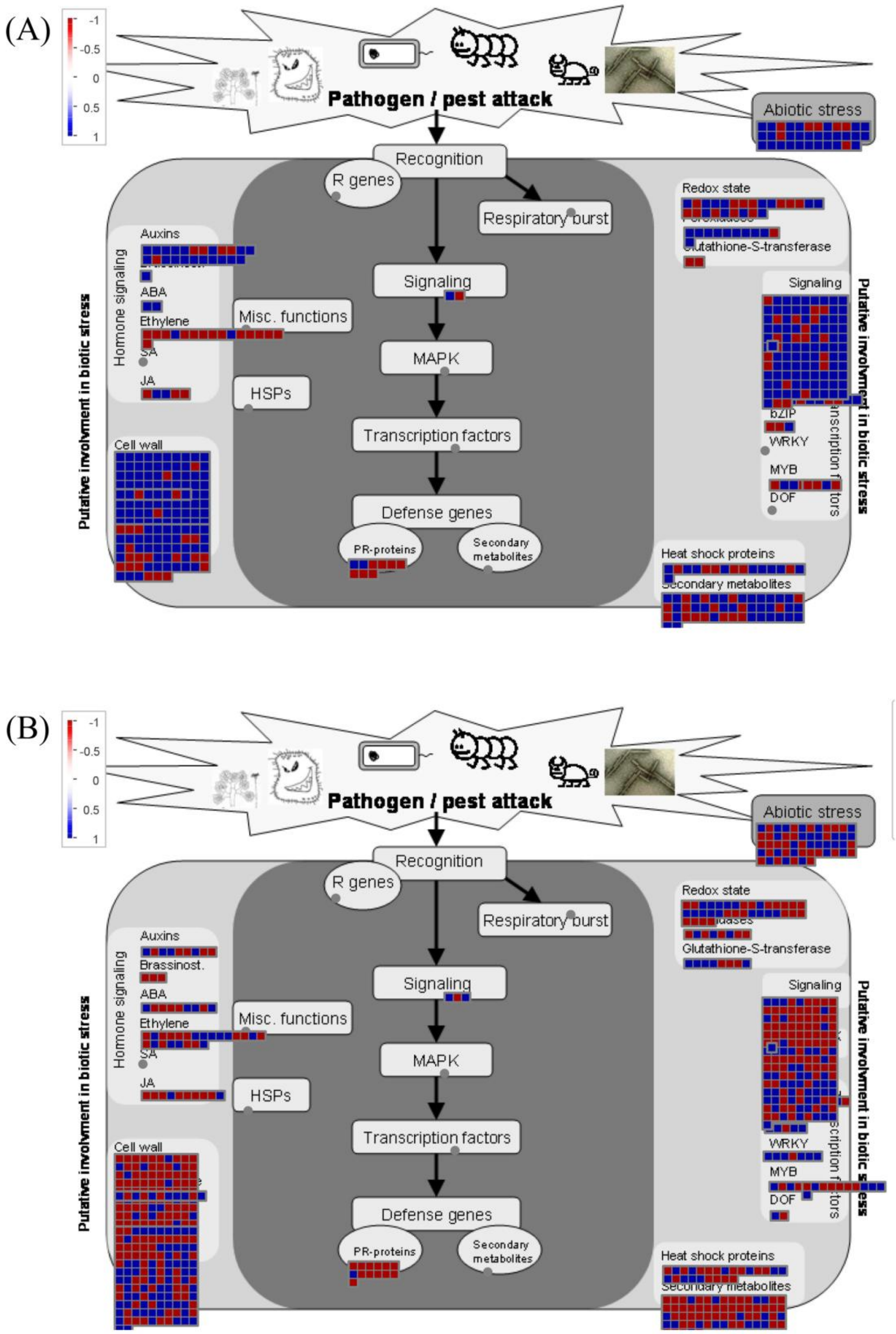

Figure S4. MapMan software output to provide an overview of the effect of scions (A) and rootstocks (B) induced on biotic stress responses. Up-regulated and down-regulated transcripts are shown in red and blue, respectively.
